# Serum Fibroblast Growth Factor 19 and Total Bile Acid Concentrations Are Potential Biomarkers of Hepatocellular Carcinoma in Patients with Type 2 Diabetes Mellitus

**DOI:** 10.1155/2020/1751989

**Published:** 2020-02-11

**Authors:** Yi Sun, Minxia Zhu, Hui Zhao, Xiaoqing Ni, Renan Chang, Jianyou Su, Hua Huang, Shiwei Cui, Xinlei Wang, Jin Yuan, Jie Yuan, Rong OuYang, Rongping Zhang, Wei Chen, Yunjuan Gu, Yezi Sun

**Affiliations:** ^1^Department of Endocrinology and Metabolism, Affiliated Hospital of Nantong University, Nantong, Jiangsu, China; ^2^Department of Endocrinology and Metabolism, Wuxi Xishan People's Hospital, Wuxi, Jiangsu, China; ^3^Department of Interventional Radiography, Affiliated Hospital of Nantong University, Nantong, Jiangsu, China; ^4^Department of Geriatrics, Affiliated Hospital of Nantong University, Nantong, Jiangsu, China; ^5^Department of Hepatopancreatobiliary Surgery, Affiliated Hospital of Nantong University, Nantong, Jiangsu, China; ^6^Department of Laboratory Medicine, Affiliated Hospital of Nantong University, Nantong, Jiangsu, China; ^7^Department of Pathology, Affiliated Hospital of Nantong University, Nantong, Jiangsu, China; ^8^Department of Endocrinology and Metabolism, Zhangjiagang First People's Hospital, Suzhou, Jiangsu, China

## Abstract

**Purpose:**

Type 2 diabetes mellitus (T2DM) carries a high risk of hepatocellular carcinoma (HCC). Both serum fibroblast growth factor 19 (FGF19) and bile acid concentrations are associated with T2DM and HCC. We aimed at evaluating the relationships between FGF19 and bile acid concentrations and HCC in patients with T2DM.

**Methods:**

Twenty-seven healthy volunteers (control group), 27 patients with T2DM (T2DM group), 16 patients with newly diagnosed HCC (HCC group), and 10 T2DM patients with newly diagnosed HCC (T2DM-HCC group) were studied at the Affiliated Hospital of Nantong University between June 2016 and June 2017. The serum concentrations of serum FGF19 and total bile acids (TBA) were measured in all the participants. Correlation analysis and multiple stepwise regression analysis of the FGF19 and TBA concentrations were performed in all the participants and in the four groups.

**Results:**

The concentrations of FGF19 were 220.5 pg/ml, 185.1 pg/ml, 115.8 pg/ml, and 70.4 pg/ml in the HCC, T2DM-HCC, control, and T2DM groups, respectively (*p* < 0.001), and the TBA concentrations were 21.75 *μ*mol/l, 14.25 *μ*mol/l, 14.25 *μ*mol/l, 14.25 *μ*mol/l, 14.25 *p* < 0.001), and the TBA concentrations were 21.75 *r* = 0.777; *p* < 0.001), and the TBA concentrations were 21.75 *r* = 0.777; *p* < 0.001), and the TBA concentrations were 21.75 *r* = 0.777; *p* < 0.001), and the TBA concentrations were 21.75 *r* = 0.777; *p* < 0.001), and the TBA concentrations were 21.75 *r* = 0.777; *p* < 0.001), and the TBA concentrations were 21.75

**Conclusions:**

Simultaneous increase of serum FGF19 and TBA levels may be used as indicators of HCC screening at early stage in patients with T2DM.

## 1. Introduction

Diabetes and cancer are common health problems worldwide. Epidemiologic studies have shown that people with diabetes, especially those with type 2 diabetes, have a significantly higher risk of cancer [1, 2]. Specifically, a meta-analysis suggested that diabetes increases the risk of liver and pancreatic cancer [3], and a majority of studies have also shown that diabetes is an independent risk factor for the development and progression of HCC [4, 5]. Interestingly, both fibroblast growth factor (FGF) 19 and bile acids play important roles in the pathogenesis of T2DM and HCC [6–8].

The FGF family contains 23 members, which achieve their effects by binding to tyrosine kinase receptors, including the FGF receptor (FGFR) [9]. FGF19 has been the subject of much recent attention and is a member of the “endocrine” subgroup of the FGF superfamily [10]. Previous studies have shown that FGF19 has an insulin-like regulatory function in metabolism, and a low FGF19 concentration or impairment in FGF19 signaling can lead to glucose metabolism disorders in insulin-resistant and T2DM patients [11, 12]. In addition, high FGF19 concentrations have been reported to be present in HCC patients and are associated with a poor prognosis [8]. Furthermore, the FGF19-FGFR4 pathway may be a key contributor to the pathogenesis of HCC [13]. Because of its apparently contrasting role in these two diseases, a genetically modified FGF19 (M70) has been developed that retains its beneficial metabolic activity but lacks the deleterious mitogenic activity and is currently undergoing clinical trials [14, 15].

Bile acids are synthesized from cholesterol in the liver, conjugated to glycine or taurine, actively secreted into bile, stored in the gall bladder, and then released into the intestine following the ingestion of a meal [16]. Previous studies have suggested that bile acid metabolism is associated with glucose metabolism and liver cancer: patients with T2DM have higher serum concentrations of bile acids than healthy individuals [17] and high concentrations of bile acids can promote the development and progression of human HCC [18, 19].

However, the physiologic roles of FGF19 and bile acids in patients with T2DM and HCC patients remain poorly understood. Therefore, we aimed at evaluating the relationships of the concentrations of FGF19 and bile acids with HCC and T2DM in patients, with the intention of determining whether they might represent useful markers of HCC in T2DM patients.

## 2. Materials and Methods

### 2.1. Participants

Twenty-seven healthy volunteers (control group), 27 patients with T2DM (T2DM group), 16 patients with newly diagnosed HCC (HCC group), and 10 T2DM patients with newly diagnosed HCC (T2DM-HCC group) were recruited at the Affiliated Hospital of Nantong University between June 2016 and June 2017. The inclusion criteria were as follows: for the T2DM group, a recent diagnosis and no use of hypoglycemic drugs; for the HCC group, a recent diagnosis and no surgery or chemotherapy; and for the T2DM-HCC group, a diagnosis of HCC ≥ 1 year after a diagnosis of T2DM, with no surgery or chemotherapy. The exclusion criteria were as follows: type 1 diabetes; special types of diabetes; secondary diabetes; liver, kidney, or cardiac dysfunction; chronic pancreatitis, thyroiditis, rheumatism, or other chronic diseases; acute complications of diabetes; the use of drugs such as glucocorticoids or antibiotics; mental illness; viral hepatitis; pregnancy or lactation; long-term alcohol abuse; and a body mass index (BMI) ≥30 kg/m^2^.

A total of 80 Chinese people (60 men and 20 women; 18–75 years) participated in the present study. All the patients were assessed by the collection of a detailed medical history (including sex, age, smoking and drinking habits, and daily medication) and physical examination. The study was approved by the Ethics Committee of the Affiliated Hospital of Nantong University, and each participant provided their written informed consent.

The following data were collected from clinical records: sex; age; anthropometric parameters; including height, body mass, and waist circumference (WC); and blood pressure. BMI was calculated as body mass/height^2^ (kg/m^2^). Blood samples were obtained from each participant following an overnight fast of 12 h. Venous blood samples were collected in clot-activator tubes and centrifuged at 3,000 ×g for 10 min to obtain serum, which was stored at −80 C and analyzed within 3 months.

### 2.2. Laboratory Measurements

Plasma glucose concentration was measured using the glucose oxidase method. Serum insulin concentration was measured by radioimmunoassay (Beijing North Institute of Biological Technology, Beijing, China), according to the manufacturer's instructions. Glycosylated hemoglobin (HbA1c) was measured by high-performance liquid chromatography (Bio-Rad Laboratories, Hercules, CA). The following indices were measured using standard methods on a parallel, multichannel analyzer (Hitachi 7600-020, Tokyo, Japan): a liver panel, comprising aspartate aminotransferase (AST), alanine aminotransferase (ALT), *γ*-glutamyl transferase (*γ*GT), alkaline phosphatase (ALP), and albumin (ALB); a renal function panel, comprising serum creatinine (Cr), cystatin-C (Cys-C), and uric acid (Ua); a lipid panel, comprising total cholesterol (TC), triacylglycerol (TG), high-density lipoprotein (HDL), and low-density lipoprotein (LDL) cholesterol; and total bile acids (TBA). Serum alpha-fetoprotein (AFP) and carcinoembryonic antigen (CEA) concentrations were measured using chemiluminescence immunoassays. Basal insulin secretion and sensitivity were assessed using the homeostasis model assessment of insulin secretion (HOMA-%B) and insulin resistance (HOMA-IR) [20]. Estimated glomerular filtration rate (eGFR) was calculated using the Modification of Diet in Renal Disease equation: eGFR (ml/min/1.73 m^2^) = 186 × creatinine^−1.154^ × age^−0.203^ (×0.742 for women) [21].

Serum FGF19 concentration was determined using enzyme-linked immunosorbent assay (ELISA) kits (Antibody and Immunoassay Services, University of Hong Kong). This assay has been shown to be highly specific for human FGF19 and not to demonstrate cross-reactivity with other members of the FGF family. The intra-assay and interassay coefficients of variation were 4.5% and 5.6%, respectively.

### 2.3. Diagnosis and Definitions

T2DM was diagnosed according to the 1999 diagnostic criteria of the World Health Organization (WHO) [22]. The diagnosis of HCC was based on the guidelines of the European Association for the Study of the Liver [23].

### 2.4. Statistical Analysis

Data were analyzed using SPSS v.22.0 statistical software (IBM, Inc., Armonk, NY, USA). Categorical data are expressed as frequencies (percentages) and were compared using the chi-square test. Normally distributed data are expressed as the mean ± standard deviation (SD), and data that were not normally distributed, according to the Kolmogorov–Smirnov test, were logarithmically transformed before analysis and are expressed as the median (interquartile range). If data were still not normally distributed after transformation, they were analyzed using the Kruskal–Wallis *H* test. The independent-samples *t*-test was used for comparisons between two groups and one-way ANOVA was used for comparisons between multiple groups. Pearson and Spearman correlation analysis and multiple regression analysis were used to characterize the relationships between FGF19 and other variables. *p* < 0.05 was considered to represent statistical significance.

## 3. Results

**Figure 1:**
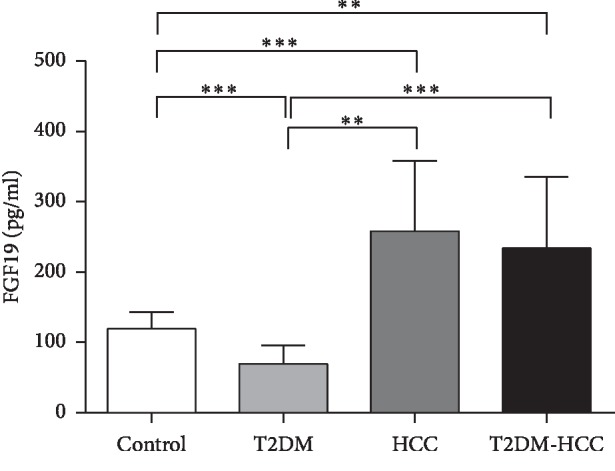
Comparison of fasting serum FGF19 levels in different groups. ^*∗∗*^*p* < 0.01；^*∗∗∗*^*p* < 0.001.

**Figure 2:**
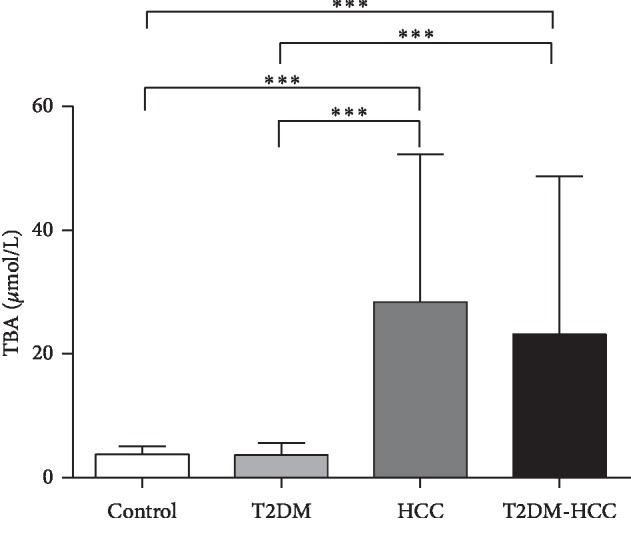
Comparison of total bile acid levels in different groups. ^*∗∗∗*^*p* < 0.001.

As shown in Table 1, there were no significant differences in sex, blood pressure, Cr, Ua, or eGFR among the four groups. The T2DM & HCC patients were older than the control group individuals (*p* < 0.05). When compared with the control group, the T2DM group had significantly higher concentrations of fasting plasma glucose (FPG) and CEA and lower concentrations of FGF19 (all *p* < 0.05). The subjects with HCC or T2DM & HCC had significantly higher *γ*GT, ALP, TBA, AFP, CEA, and FGF19 and lower ALB, HDL, and LDL levels than did control group subjects (all *p* < 0.05). In addition, when compared with the T2DM group, the patients with HCC or T2DM & HCC also had higher *γ*GT, TBA, AFP, and FGF19 concentrations (all *p* < 0.05) (Figures 1 and 2).

**Figure 3:**
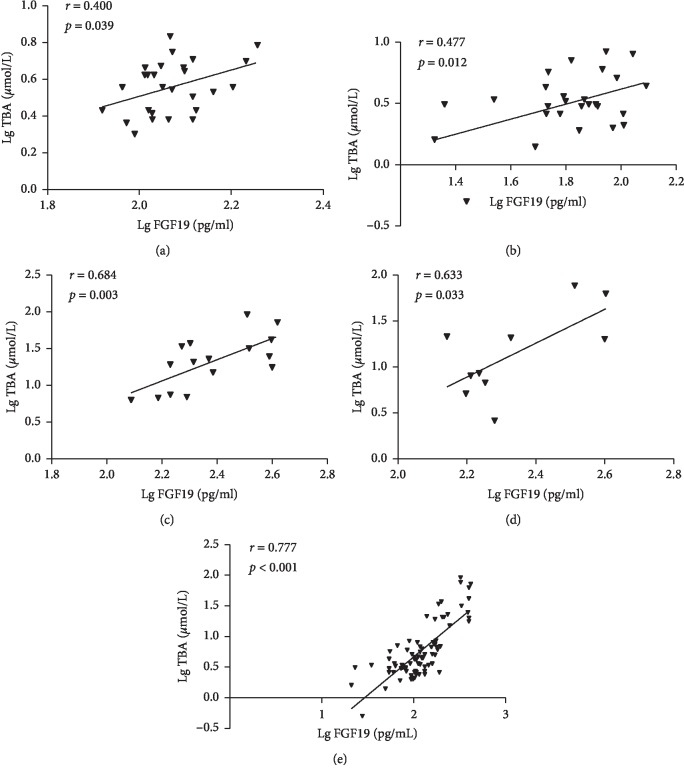
Correlations of fasting FGF19 concentration with fasting total bile acid level in control (a), T2DM (b), HCC (c), T2DM-HCC (d), and total population (e).

Correlation analysis showed that Lg FGF19 was significantly positively correlated with TBA in each group (all *p* < 0.05; Table 2 and Figure 3). In the total population and the HCC group, Lg FGF19 was positively correlated with AFP (all *p* < 0.05, Table 2).

Multiple stepwise linear regression analysis showed that the FGF19 and TBA concentrations were correlated in each group (Table 3), and this correlation remained after adjustment for age, SBP, FPG, and CEA (Table 3).

## 4. Discussion

### 4.1. Relationships between FGF19, Glucose Metabolism, and HCC

FGF19 is a metabolic regulator that is produced primarily by cells in the distal ileum and acts on the liver [9]. A number of studies have shown that FGF19 plays an important role in glucose and lipid metabolism. Barutcuoglu et al. found that FGF19 is negatively associated with TG and HbA1c in diabetic patients with metabolic syndrome [24]. Recently, another study reported that the concentration of FGF19 in obese patients increases after successful weight loss following bariatric surgery [25]. Furthermore, animal experiments have demonstrated that the administration of FGF19 to mice can rapidly improve glucose metabolism without the necessity for weight loss [26], probably because it increases fatty acid oxidation and metabolic rate [27].

Previous studies have shown that the expression of FGF19 is significantly higher in patients with liver cirrhosis and liver cancer and is significantly associated with the pathologic stage of liver cancer [8, 13]. Furthermore, Kang et al. demonstrated that a unique molecular subtype of FGF19 is associated with poor prognosis in liver cancer [28]. Finally, overexpression of FGF19 in the skeletal muscle of mice results in liver dysplasia and HCC [29]. However, to our knowledge, the present study is the first to measure the concentrations of FGF19 in patients with T2DM and HCC. We compared the FGF19 concentrations in the control, T2DM, HCC, and T2DM-HCC groups and found that they were high in both T2DM-HCC and HCC patients, but low in T2DM patients (Table 1 and Figure 1). These results suggest that FGF19 may have beneficial effects on metabolism but promote HCC development.

### 4.2. Mechanisms of the Effects of FGF19 on Glucose Metabolism and HCC

The mechanisms of the effects of FGF19 on metabolism have been thoroughly studied. Zhang et al. demonstrated that a reduction in FGF19 concentration has effects on glucose effectiveness and hepatic glucose production, which leads to an increase in fasting blood glucose [6]. FGF15/19 have additive effects to insulin to reduce hepatic gluconeogenesis by inhibiting the cAMP-response element-binding protein-peroxisome proliferator-activated receptor coactivator-1a signaling cascade after a meal [12]. Moreover, FGF19 activates an insulin-independent pathway to regulate the synthesis of liver proteins and glycogen [11]. In addition, central injection of FGF19 has a hypoglycemic effect because it increases the non-insulin-dependent glucose processing capacity in the brain [30] and can also improve peripheral insulin signaling by inducing extracellular signal-related kinase (ERK) 1/2 signaling in the hypothalamus [31].

Lee et al. reported that FGF19 is secreted by cells in the tumor microenvironment and acts on tumor and stromal cells in autocrine and paracrine fashion [32]. Higher expression of FGF19 in HCCs promotes tumor cell survival and has antiapoptotic effects that are exerted through the FGFR4-glycogen synthase kinase (GSK)3*β*-Nrf2 signaling cascade [33]. Epithelial-mesenchymal transition (EMT) is known to be associated with tumor aggressiveness and poor survival, and Zhao et al. found that FGF19 induces EMT via the FGFR4/GSK3*β*/*β*-catenin axis in HCC cells [34]. In addition, Cui et al. reported that FGF15 binds to FGFR4 and promotes the development of HCC by activating EMT and Wnt/*β*-catenin signaling in a microenvironment in which lipid metabolism is disordered [35].

Consistent with previous findings, we have shown that HCC patients have significantly lower FPG and TC concentrations than healthy people, and a negative correlation between FGF19 concentration and HbA1c (*r* = −0.393, *p*=0.043; not shown in the table) in T2DM patients, consistent with FGF19 improving glucose and lipid metabolism. However, FGF19 concentration did not correlate with FPG or other indicators of glucose metabolism in patients with both T2DM and HCC. The explanation for this may be that the concentration of FGF19 is low in T2DM and high in HCC and has counterregulatory effects; alternatively, the sample size may have been too small or there may have been confounding factors involved. Furthermore, in line with the findings of previous studies, we have demonstrated that FGF19 positively correlates with AFP in HCC patients (Table 2).

### 4.3. Relationships between TBA, Glucose Metabolism, and HCC

Haeusler et al. found that 12*α*-hydroxylated bile acid concentrations positively correlate with insulin resistance in healthy individuals and that T2DM patients have higher TBA concentrations than healthy people [17]. Specifically, when compared with healthy people, patients with T2DM were found to have higher concentrations of lithocholic acid and taurocholic acid, but lower concentrations of ursodeoxycholic acid [36]. In addition, the proportion of 12*α*-hydroxylated bile acids was found to be higher in diabetic rats and mice [37, 38].

Li et al. reported that glucose and insulin can induce CYP7A1 gene expression and bile acid synthesis [38], and Dufer et al. demonstrated that sodium taurochenodeoxycholate stimulates insulin secretion in beta-cells by activating farnesoid X receptor (FXR) and inhibiting K-ATP channels [39]. In addition, studies in rats with T2DM have shown that such changes in bile acid concentrations have a negative impact on glucose metabolism by inhibiting bile acid receptor TGR5/FXR-mediated pathways in the colon, liver, and pancreas [17]. A previous study has also shown that prolonged exposure to bile acids may promote the development and progression of HCC in cholestatic liver disease [19]. Furthermore, feeding mice with a bile acid-rich diet increases bile acid concentrations and promotes N-nitrosodiethylamine-induced liver tumorigenesis [18].

With regard to the mechanism involved, Yamada et al. reported that the intestinal microbiome plays a major role in the conversion of primary to secondary bile acids, such as deoxycholic acid, which promote the development of nonalcoholic steatohepatitis-associated HCC in mice by activating mechanistic target of rapamycin (mTOR) signaling in hepatocytes [40]. Moreover, one of the most important bile acids, glycine chenodeoxycholic acid, has been reported to induce autophagy through the AMP-activated protein kinase/mTOR signaling pathway, which promotes the invasiveness of HCC cells [41]. Consistent with these findings, we have shown that TBA concentrations are higher in patients with HCC and in those with both T2DM and HCC. All these findings suggest that bile acid concentrations should be strictly controlled. However, we also found that T2DM patients have slightly lower TBA concentrations, which is not consistent with the results of previous studies, although we did not measure the concentrations of individual bile acids, so we cannot draw conclusions with regard to the importance of the concentrations of 12*α*-hydroxylated bile acids.

### 4.4. The Relationship between FGF19 and TBA Concentrations

Previous studies have demonstrated that bile acids can upregulate the synthesis of FGF19 and FGF19 can reduce bile acid synthesis and control gall bladder volume [42, 43]. Holt et al. found that circulating bile acid concentrations increase after a meal, which activates FXR to induce the production and secretion of FGF19, reducing the bile acid concentrations [44]. Recently, Zhang et al. reported that a reduction in chenodeoxycholic acid results in a lower FGF19 concentration, an effect that is mediated through FXR [45]. In addition, some previous studies have shown that the binding of FGF15/19 to FGFR4 reduces the expression of CYP7A1, thereby inhibiting the synthesis of bile acids [46, 47]. Finally, multiple pathways have been reported to be involved in the regulation of bile acids by FGF19, including the ERK [48], c-Jun N-terminal kinase [44], and phosphatidylinositol 3-kinase [49] pathways.

Our results are consistent with these findings because the concentrations of FGF19 and TBA were higher in the T2DM-HCC and HCC groups than in the control and T2DM groups (Figures 1 and 2). In addition, we have shown that the FGF19 concentration positively correlates with that of TBA (Figure 3), and this association remains after multiple stepwise regression analysis (Table 3). It has been suggested that FGF19 may play an important role in the regulation of bile acid homeostasis. Thus, both FGF19 and bile acids may be involved in the development of HCC in T2DM patients, and they may be useful as markers of this disease.

The present study had a number of limitations. First, the sample size was relatively small; second, this was a single-center retrospective case-control study; and third, diabetic duration, time of antidiabetic drugs applied, and interaction of drugs are unavoidable confounding factors, which are difficult to eliminate or to adjust. Therefore, larger scale multicenter prospective studies are needed to validate our findings and to identify the underlying mechanisms of the associations identified.

## 5. Conclusions

In conclusion, the simultaneous changes of serum FGF19 and TBA levels may be used as indicators of HCC screening at early stage in patients with T2DM.

## Figures and Tables

**Table 1:** Anthropometric parameters and biochemical indexes among subjects with control, T2DM, HCC, and T2DM-HCC.

Variables	Control (*n* = 27)	T2DM (*n* = 27)	HCC (*n* = 16)	T2DM-HCC (*n* = 10)	*p*
Male/female	21/6	20/7	10/6	9/1	0.448
Age (years)	49.81 ± 8.06	50.52 ± 12.41	55.38 ± 8.29	62.60 ± 9.06^#‡^	0.004
BMI (kg/m^2^)	23.64 ± 2.30	25.22 ± 2.51	22.62 ± 3.39^‡^	25.16 ± 2.53	0.010
SBP (mmHg)	125 ± 7	129 ± 14	124 ± 13	134 ± 14	0.112
DBP (mmHg)	76 ± 10	78 ± 11	77 ± 10	73 ± 7	0.507
FPG (mmol/L)	5.20 (4.90–5.40)	8.80 (7.40–10.70)^#^	4.45 (4.13–4.98)^#‡^	7.30 (6.05–10.73)^§^	<0.001
ALT (*μ*/L)	26 (17–36)	35 (20–54)	51 (31–90)^#^	38 (28–87)	0.014
*γ*GT (*μ*/L)	27 (20–35)	29 (20–47)	114 (50–218)^#‡^	126 (72–256)^#‡^	<0.001
ALP (*μ*/L)	68 (58–84)	80 (62–101)	121 (92–164)^#^	144 (94–241)^#^	<0.001
ALB (g/L)	45.70 (43.90–46.30)	41.70 (38.90–44.30)^#^	37.30 (35.08–39.80)^#‡^	34.70 (31.08–36.90)^#‡^	<0.001
TBA (*μ*mol/l	3.60 (2.70–4.60)	3.10 (2.60–4.40)	21.75 (9.28–36.25)^#‡^	14.25 (6.30–31.60)^#‡^	<0.001
TBIL (*μ*mol/l)	14.5 (11.3–17.0)	15.1 (12.7–19.0)	17.7 (13.9–22.3)	21.7 (12.7–27.3)	0.046
DBIL (*μ*mol/l)	3.7 (3.2–4.5)	4.3 (3.4–6.5)	5.6 (4.7–6.6)^#^	7.3 (4.6–9.4)	0.001
Cr (*μ*mol/l)	60.75 ± 12.05	58.26 ± 11.94	57.94 ± 13.49	60.5 ± 24.06	0.935
Ua (*μ*mol/l)	292.00 (221.00–319.00)	265.00 (210.00–305.00)	293.50 (231.75–356.75)	235.00 (177.00–392.50)	0.651
Cys-C (mg/l)	0.70 (0.70–0.80)	0.70 (0.60–0.70)	0.85 (0.80–0.98)^‡^	0.85 (0.70–1.13)	<0.001
eGFR (ml/min/1.73 m^2^)	90.07 (82.73–102.60)	102.45 (82.84–117.12)	97.83 (80.77–121.89)	106.54 (75.37–137.62)	0.774
TC (mmol/l)	4.84 ± 0.51	5.25 ± 0.90	3.88 ± 0.86^#‡^	3.66 ± 1.15^#‡^	<0.001
TG (mmol/l)	0.85 (0.76–1.21)	1.37 (0.86–2.54)	1.00 (0.64–1.25)	1.02 (0.77–1.36)	0.006
HDL (mmol/l)	1.41 ± 0.31	1.09 ± 0.27^#^	0.83 ± 0.33^#‡^	0.89 ± 0.35^#^	<0.001
LDL (mmol/l)	2.75 ± 0.48	2.95 ± 0.86	1.87 ± 0.70^#‡^	1.86 ± 0.80^#‡^	<0.001
AFP (ng/ml)	2.65 (2.13–4.10)	3.50 (2.57–4.17)	604.31 (413.930–2039.87)^#‡^	671.08 (422.58–10000)^#‡^	<0.001
CEA (ng/ml)	1.20 (0.90–1.50)	2.00 (1.40–3.20)^#^	2.05 (1.55–3.00)^#^	2.25 (0.90–2.98)^#^	<0.001
FGF19 (pg/ml)	115.78 (104.24–130.68)	70.44 (53.51–88.20)^#^	220.53 (174.21–373.54)^#‡^	185.07 (161.47–344.14^)^^#‡^	<0.001

Data are mean ± standard deviations (SD) or median (interquartile range). Control: healthy control; T2DM: newly diagnosed type 2 diabetes mellitus patients; HCC: newly diagnosed hepatocellular carcinoma patients; T2DM-HCC: type 2 diabetes mellitus combined newly diagnosed hepatocellular carcinoma patients. BMI: body mass index; SBP: systolic blood pressure; DBP: diastolic blood pressure; FPG: fasting plasma glucose; ALT: alanine aminotransferase; GGT: gamma-glutamyl transferase; ALP: alkaline phosphatase; ALB: albumin; TBA: total bile acids; TBIL, total bilirubin; DBIL, direct bilirubin; Cr: creatinine; Ua: uric acid; Cys-C: cystatin-C; TC: total cholesterol; TG: triglyceride; HDL: high-density lipoprotein cholesterol; LDL: low-density lipoprotein cholesterol; AFP: alpha-fetoprotein; CEA: carcinoembryonic antigen; FGF19: fibroblast growth factor 19; eGFR: estimated glomerular filtration rate. *x*^2^ test for gender comparison; one-way analysis of variance (ANOVA) for normally distributed variables; Kruskal–Wallis *H* test for nonnormally distributed variables.*p* for comparisons among the four groups; ^#^*p* < 0.05, compared with control; ^‡^*p* < 0.05, compared with T2DM; ^§^*p* < 0.05, compared with HCC.

**Table 2:** Correlation analysis between Lg FGF19 and other parameters.

	Control		T2DM		HCC		T2DM-HCC	
	*r*	*p*	*r*	*p*	*r*	*p*	*r*	*p*
Age	−0.215	0.282	0.176	0.381	0.168	0.533	0.056	0.877
BMI	−0.242	0.224	0.006	0.975	0.481	0.059	−0.390	0.265
FPG	−0.273	0.168	0.047	0.815	0.162	0.550	0.395	0.259
ALT^†^	−0.152	0.451	−0.409	0.034	−0.068	0.802	0.145	0.690
*γ*GT^†^	−0.450	0.018	−0.042	0.836	0.536	0.032	0.357	0.311
ALB	−0.266	0.179	−0.371	0.057	−0.036	0.894	−0.104	0.774
TBA^†^	0.400	0.039	0.477	0.012	0.684	0.003	0.673	0.033
TBIL^†^	−0.363	0.063	−0.035	0.861	−0.103	0.704	0.010	0.978
eGFR^†^	0.424	0.027	−0.005	0.980	0.062	0.819	0.047	0.898
Cys-C^*∗*^	−0.227	0.255	0.384	0.048	−0.058	0.833	−0.300	0.400
TC	−0.485	0.010	−0.070	0.728	0.026	0.923	−0.333	0.347
TG	−0.333	0.090	−0.229	0.251	−0.220	0.413	−0.064	0.861
LDL	−0.528	0.005	0.132	0.511	0.024	0.931	−0.079	0.828
AFP^†^	0.001	0.998	0.140	0.486	0.540	0.031	0.560	0.092
CEA^†^	−0.081	0.689	−0.091	0.650	0.078	0.774	0.464	0.177

^†^Log-transformed variables; Pearson correlation analysis for normally distributed variables; ^*∗*^Spearman correlation analysis for nonnormally distributed variables; control: healthy control; T2DM: newly diagnosed type 2 diabetes mellitus patients; HCC: newly diagnosed hepatocellular carcinoma patients; T2DM-HCC: type 2 diabetes mellitus combined newly diagnosed hepatocellular carcinoma patients. Total: total population. Abbreviations are same as in Table 1. *p* values <0.05 were considered significant.

**Table 3:** Multivariable regression analysis of various biomarkers versus FGF19.

	Control		T2DM		HCC		T2DM-HCC	
	Standard *β*	*p*	Standard *β*	*p*	Standard *β*	*p*	Standard *β*	*p*
Model 1								
TBA	0.397	0.040	0.400	0.039	0.562	0.024	0.701	0.024
Model 2								
TBA	0.414	0.031	0.461	0.032	0.637	0.037	0.826	0.044

Model 1: crude model without covariate adjustment; model 2: adjustment for age, SBP, FPG, and CEA. Control: healthy control; T2DM: newly diagnosed type 2 diabetes mellitus patients; HCC: newly diagnosed hepatocellular carcinoma patients; T2DM-HCC: type 2 diabetes mellitus combined newly diagnosed hepatocellular carcinoma patients. Abbreviations are same as in Table 1.

## Data Availability

The data used to support the findings of this study are available from the corresponding author upon request.
